# Metatranscriptomic Analysis Reveals Actively Expressed Antimicrobial-Resistant Genes and Their Hosts in Hospital Wastewater

**DOI:** 10.3390/antibiotics13121122

**Published:** 2024-11-23

**Authors:** Yusuke Ota, Fei Chen, Isaac Prah, Samiratu Mahazu, Kimiyo Watanabe, Teruaki Kinoshita, Yoshiaki Gu, Yoko Nukui, Ryoichi Saito

**Affiliations:** 1 Department of Molecular Microbiology and Immunology, Institute of Science Tokyo, Tokyo 113-8510, Japan; y-ota.micr@tmd.ac.jp (Y.O.);; 2 Department of Pharmaceutical and Environmental Sciences, Tokyo Metropolitan Institute of Public Health, Tokyo 169-0073, Japan; 3 Department of Infectious Diseases, Institute of Science Tokyo, Tokyo 113-8510, Japan; 4 Department of Infection Control and Laboratory Medicine, Kyoto Prefectural University of Medicine, Tokyo 602-8566, Japan

**Keywords:** antimicrobial resistance, metatranscriptomic analysis, hospital wastewater

## Abstract

Antimicrobial resistance is a major global concern and economic threat, necessitating a reliable monitoring approach to understand its frequency and spread via the environment. Hospital wastewater serves as a critical reservoir for antimicrobial-resistant organisms; however, its role in resistance gene distribution and dissemination remains poorly understood. This study integrates metagenomic and metatranscriptomic analyses, elucidating the dynamics of antimicrobial resistance in hospital wastewater. Integrated metagenomic and metatranscriptomic sequencing were used to identify actively expressed antimicrobial-resistant genes and antimicrobial-resistant bacteria, offering comprehensive insights into antimicrobial resistance dynamics in hospital wastewater. Liquid chromatography–tandem mass spectrometry analysis revealed the presence of ampicillin, sulbactam, levofloxacin, sulfamethoxazole, and trimethoprim in the sample, which could apply selective pressure on antimicrobial resistance gene expression. While multidrug resistance genes were the most prevalent sequences in both metagenome-assembled genomes and plasmids, plasmid-derived sequences showed a high mRNA/DNA ratio, emphasizing the presence of functionally expressed antimicrobial resistance genes on plasmids rather than on chromosomes. The metagenomic and metatranscriptomic analyses revealed *Serratia nevei* MAG14 with high mRNA levels of antimicrobial resistance genes; moreover, multidrug-resistant *Serratia* sp., genetically related to MAG14, was isolated from the wastewater, supporting the phenotypic characterization of crucial antimicrobial-resistant bacteria and validating the genome analysis results. The findings underscore key genes and bacteria as targets for antimicrobial resistance surveillance in hospital wastewater to protect public and environmental health.

## 1. Introduction

The emergence and spread of antimicrobial resistance (AMR) are major threats to human health and the global economy [1]. Utilizing a “One Health Approach” that encompasses humans, animals, and the environment is crucial for effectively understanding and addressing the development and dissemination of AMR, recognizing its interconnected nature and the necessity for comprehensive surveillance strategies [2]. Among AMR diffusion pathways, the aquatic environment serves as a critical hotspot for the maintenance of antimicrobial-resistant bacteria (ARB) and antimicrobial-resistant genes (ARGs) [3]. The widespread use of antibiotics in healthcare facilities is recognized as a substantial source of pollution. The accumulation of high concentrations of antibiotic compounds in sewage creates selective pressure on ARG expression and generates pathogenic ARGs, which are subsequently transferred to human communities [3]. Although a wide range of ARGs have been identified worldwide in hospital wastewater [4,5,6], the link between ARB and ARGs and their potential roles is not fully understood, necessitating effective monitoring and control of AMR in the environment.

High-throughput sequencing-based metagenomic analysis offers advantages over conventional culture-based methods and provides more comprehensive profiles of ARGs in environmental samples [7]. Plasmids play a crucial role in the horizontal transfer of ARGs among microorganisms. Recent metagenomic analyses have directly illustrated the relationship between ARGs and plasmids, providing valuable insights into the potential transfer of ARGs in environmental compartments [8]. Additionally, the metagenomic approach can demonstrate co-occurrence patterns between ARGs and their putative host organisms within microbial communities by constructing a metagenome-assembled genome (MAG) [8]. However, relying solely on metagenomics while overlooking stimulated ARGs may not elucidate bacterial activity under environmental stresses, such as antibiotic pressure. Metatranscriptomics, in combination with metagenomics, enables the simultaneous characterization of both community and gene expression, offering an extensive perspective on dynamic environments [9]. This approach reveals actively expressed ARGs in aquatic settings, elucidating water resistome profiles and the effectiveness of metatranscriptomic data for AMR monitoring [10,11]. Nevertheless, there is a lack of metatranscriptomic research focusing on hospital wastewater with high amounts of antibiotics and AMR [4]. Thus, the distribution of transcribed ARB and plasmid-derived ARGs involved in understanding their transmission remains largely unknown.

In the present study, we utilized an integrated analysis of metagenomic and metatranscriptomic sequencing to offer comprehensive insights into active ARGs among microbial communities in hospital wastewater. The selective pressures that could influence the microbial communities were initially assessed using liquid chromatography–tandem mass spectrometry (LC–MS/MS) to detect antimicrobial agents in the sample. To evaluate the distribution of AMR, ARGs in MAGs and plasmid-derived sequences were comprehensively investigated using metagenomic analysis. Subsequently, metatranscriptomics was used to produce ARG transcripts relative to the absolute amount of ARGs. Furthermore, we identified possible threats of ARB in the environment based on combined metagenomic and metatranscriptomic data and attempted to isolate them using a culture-based method for phenotypic characterization. Moreover, we explored the dynamics of AMR and the characteristics of existing key ARGs in hospital wastewater, contributing to efficient wastewater management for the protection of public health and the environment.

## 2. Results

### 2.1. Detection of Antimicrobial Agents Using LC–MS/MS

**Figure 1:**
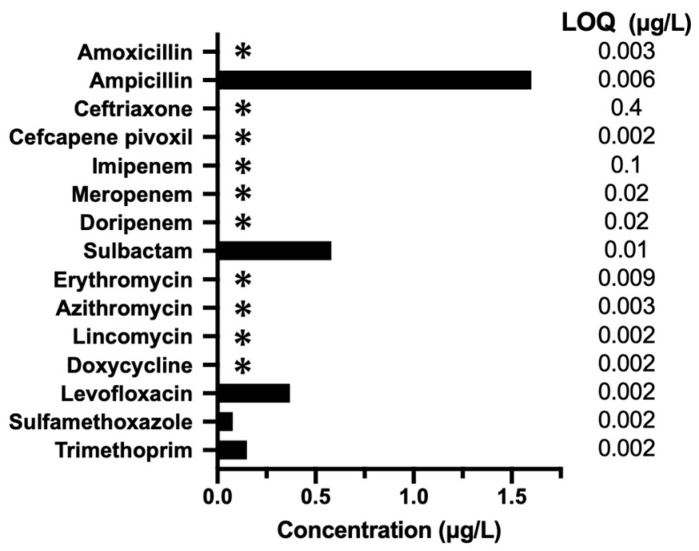
Distribution of antibiotic compounds in hospital wastewater. *: Below the limit of quantification (LOQ).

The lower limit of quantification (LOQ) for each component was determined as the concentration at which the coefficient of variation was within ±20%, as established through validity assessments (*n* = 5). The concentration of antimicrobial compounds in hospital wastewater measured using LC–MS/MS was as follows: 1.60 μg/L for ampicillin, 0.58 μg/L for sulbactam, 0.37 μg/L for levofloxacin, 0.078 μg/L for sulfamethoxazole, and 0.15 μg/L for trimethoprim; other antimicrobials were not detected (Figure 1).

### 2.2. Abundance of 16S rRNA Genes in Hospital Wastewater

**Figure 2:**
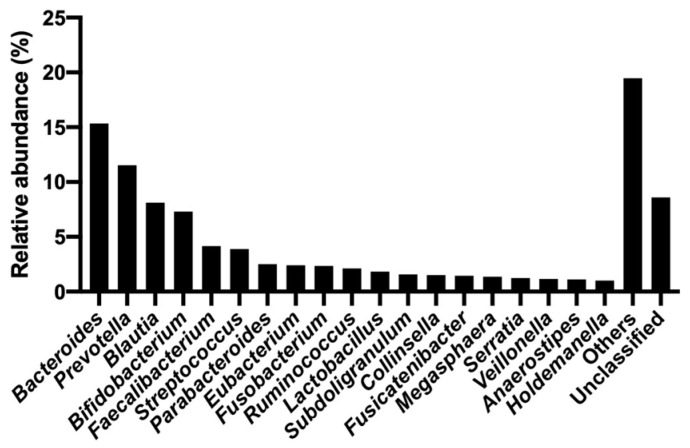
16S rRNA gene metabarcoding analysis at the genus level in hospital wastewater. Others: Genera with <1% relative abundance; Unclassified: not assigned to any taxon at the genus level.

In total, 118,286 reads were generated using 16S rRNA gene metabarcoding sequencing after quality filtering (Table S1). The taxonomic composition of the microbiota in the hospital sewage was determined by comparing it with the corresponding genera in the SILVA database [12]. In total, 153 genera were observed in the samples (Table S2). The genus *Bacteroides* was dominant, and the genera *Streptococcus* and *Serratia*, which are responsible for the largest number of deaths from AMR pathogens worldwide, were also present at a relative abundance of >1% (Figure 2).

### 2.3. Construction of MAGs and Genetic Features

The data acquired through shotgun metagenomic and metatranscriptomic sequencing are listed in Table S5. Assembly of the metagenomic data yielded 1,255,461 contigs. Mapping the reads used for assembly to the obtained contigs indicated that >90% of the reads were mapped, suggesting that most of the genome was successfully assembled. The assembled contigs were binned, and chimeric sequences were eliminated to construct 172 high-quality MAGs (Figure S1, Table S3). Taxonomic identification classified 75, 66, and 22 MAGs at the species, genus, and family levels, respectively, while 9 MAGs were not assigned to any taxon. A comparison of transcripts per kilobase million (TPM)-normalized metagenomic and metatranscriptomic data showed that the mRNA levels for 2 MAGs and 19 MAGs were 10-fold higher and 10-fold lower than those of the DNA, respectively.

**Figure 3:**
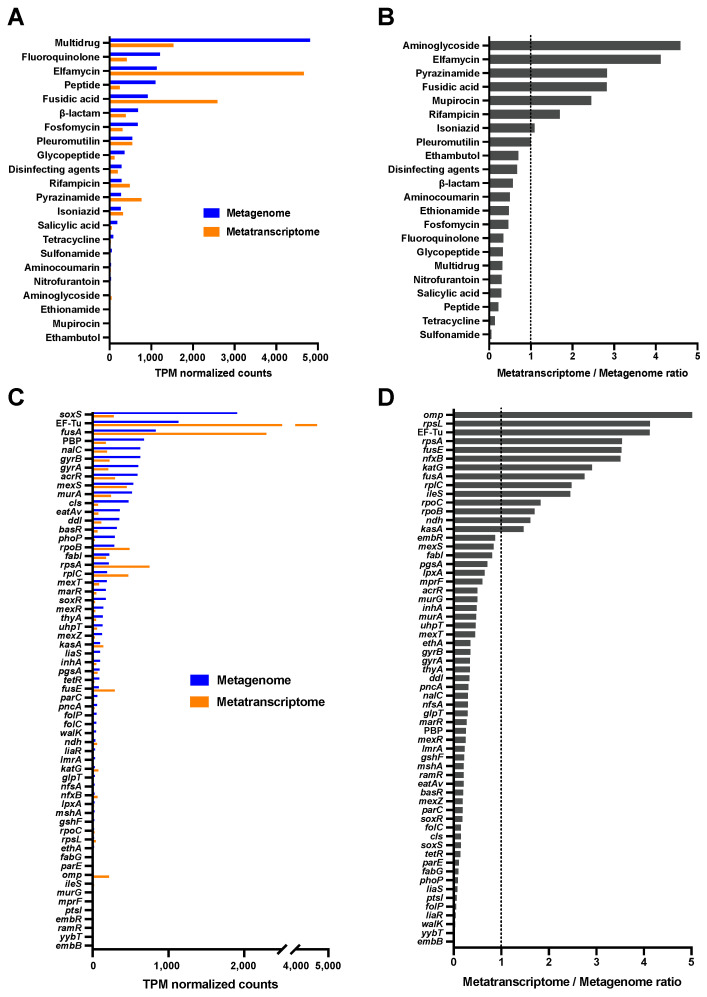
Profile of antimicrobial-resistant genes (ARGs) and transcriptional levels from metagenome-assembled genome sequences. Transcripts per kilobase million (TPM)-normalized values were calculated to compare ARG abundance. Normalized DNA and mRNA counts of drug class (**A**) and ARGs (**C**), along with their respective fold changes between expression level and gene abundance (**B**,**D**), are shown.

Based on the Comprehensive Antibiotic Resistance Database (CARD) [13], 61 ARGs associated with resistance to 22 drug classes were identified from MAG sequences (Figure 3, Table S4). Spearman correlation analysis showed the abundance of DNA and mRNA was positively correlated in both drug classes (r = 0.836) and ARGs (r = 0.808). Metagenomic data showed that ARGs associated with multidrug resistance were the most abundant, whereas elfamycin resistance genes were predominantly detected using metatranscriptomic analysis (Figure 3A). Aminoglycoside resistance genes exhibited the highest mRNA mapping counts relative to the normalized DNA (Figure 3B). For each ARG, *soxS* and EF-Tu were primarily observed in metagenomic and metatranscriptomic analyses, respectively (Figure 3C). Normalized metatranscriptome/metagenome abundance ratios were the highest for *omp* (Figure 3D).

### 2.4. Occurrence and Expression Levels of ARGs from Predicted Plasmid-Derived Sequences

**Figure 4:**
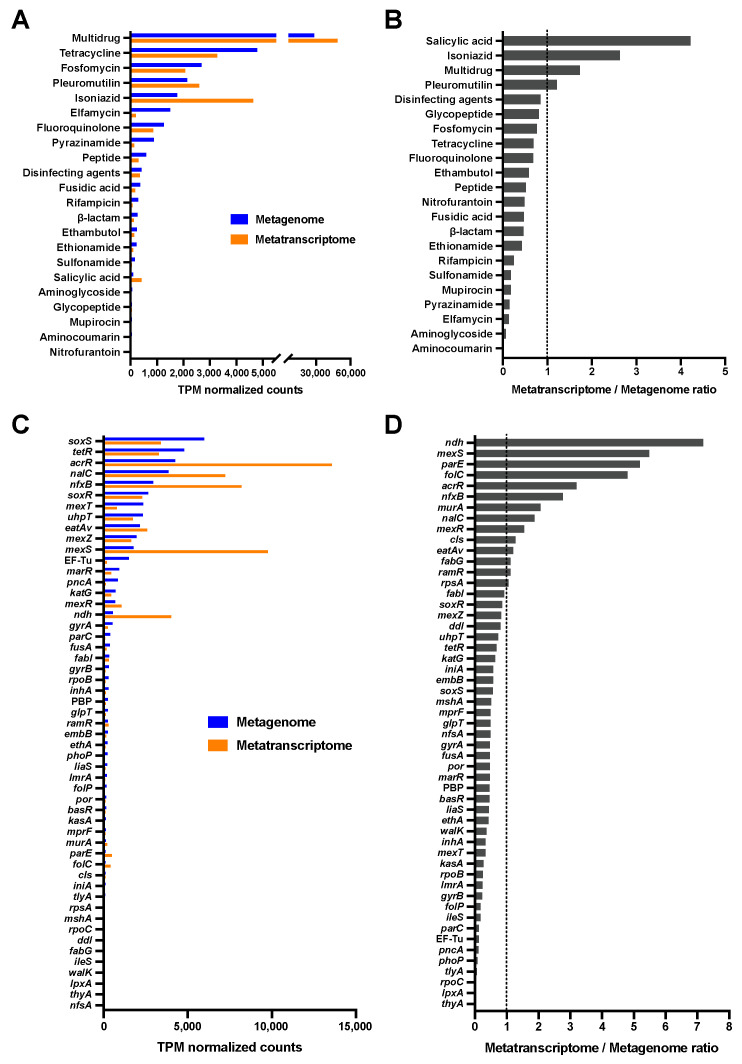
Profile of antimicrobial-resistant genes (ARGs) and transcriptional levels from predicted plasmid-derived sequences. Transcripts per kilobase million (TPM)-normalized values were calculated to compare ARG abundance. Normalized DNA and mRNA counts of drug class (**A**) and ARGs (**C**), along with their respective fold changes between expression level and gene abundance (**B**,**D**), are shown.

The classification of plasmid-derived sequences using the plasmidVerify tool and plasmid sequence database (PLSDB) resulted in the extraction of 3375 contigs of ≥1000 bp from assembled metagenomic data. Additionally, ARG prediction using CARD revealed ARGs in 1052 contigs (Table S5). The resistome analysis revealed that 53 ARGs were associated with resistance to 22 antimicrobials (Figure 4). A positive correlation was shown between DNA and mRNA abundance in drug classes (r = 0.886) and ARGs (r = 0.864). On the other hand, no correlation in mRNA/DNA ratio was observed between MAG-derived and plasmid-derived sequences (drug class; r = −0.426, ARGs; r = −0.116). In both metagenomic and metatranscriptomic analyses, ARGs related to multidrug resistance were most frequently observed in plasmid-derived sequences, whereas genes related to salicylic acid resistance were highly expressed in relative proportions (Figure 4A,B). The predominant ARGs identified in the metagenomic and metatranscriptomic data were *soxS* and *acrR*, respectively (Figure 4C). Fold changes between expression levels and gene abundance were the highest for *ndh* (Figure 4D). No relationship was found between the plasmid incompatibility groups identified using PlasmidFinder v2.0 and the possession of ARGs (Figure S2).

### 2.5. Correlations Between MAGs and ARGs

**Figure 5:**
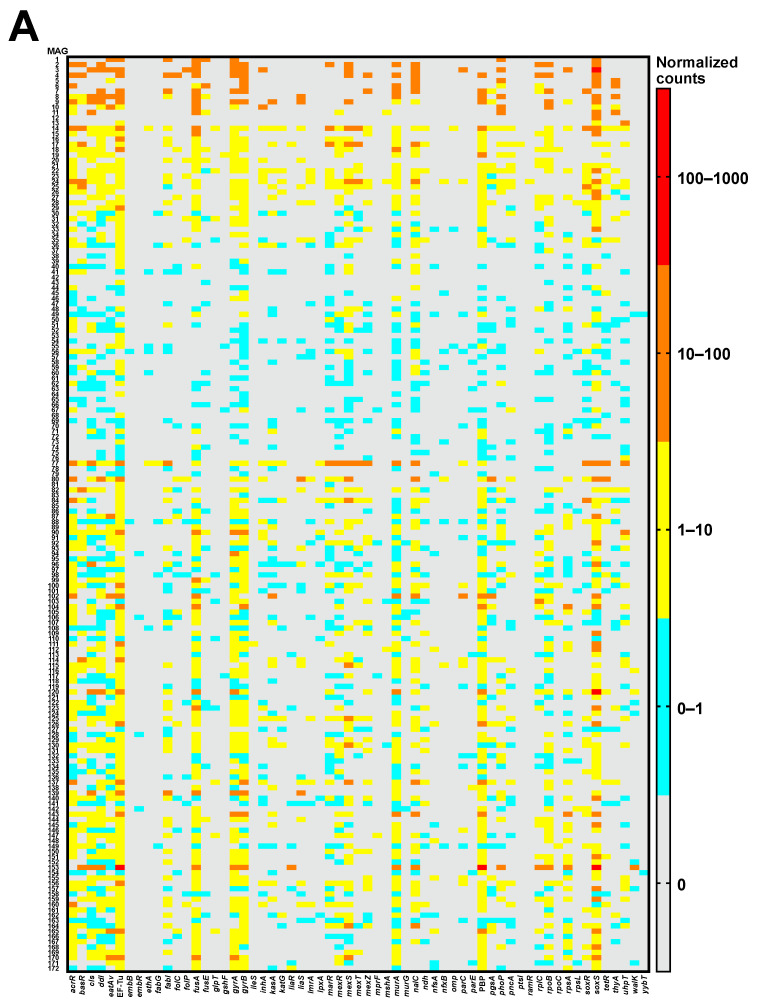

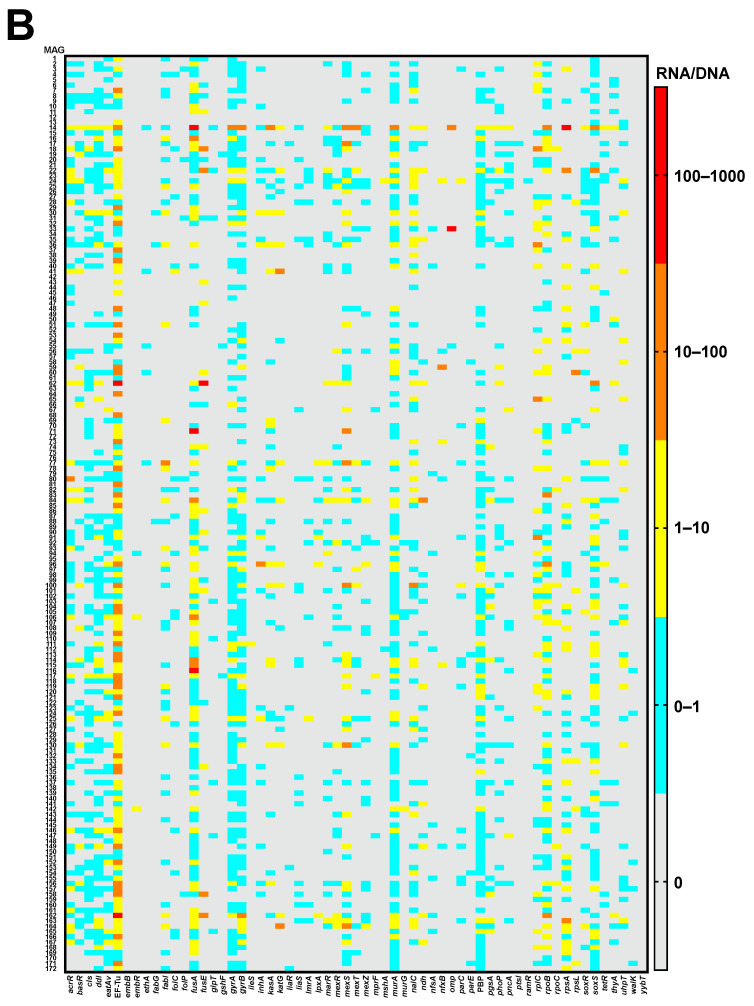
Correlation heatmap between metagenome-assembled genomes (MAGs) and antimicrobial-resistant genes (ARGs). Heatmaps, based on transcripts per kilobase million (TPM)-normalized values, illustrate ARG abundance (**A**) and fold changes between the expression level and gene abundance (**B**) for each MAG.

The abundance and expression levels of the 61 ARGs observed in the 172 MAGs are illustrated in Figure 5. The metagenomic analysis indicated increased levels of ARGs in the MAGs, with higher absolute read counts (Figure 5A, Table S3). *soxS*, which showed the highest abundance in the metagenomic data, was widely distributed in each MAG. By mapping mRNA to the normalized DNA abundance, the compensated gene expression levels were determined for each ARG (Figure 5B). Among all ARGs, there were several MAGs where EF-Tu showed >10-fold change. The bacterium MAG14, identified as *S. nevei*, exhibited the highest number of ARGs, which exhibited a >10-fold increase in expression.

### 2.6. Genetic and Phenotypic Characterization of Bacterial Isolates from Hospital Wastewater

**Figure 6:**
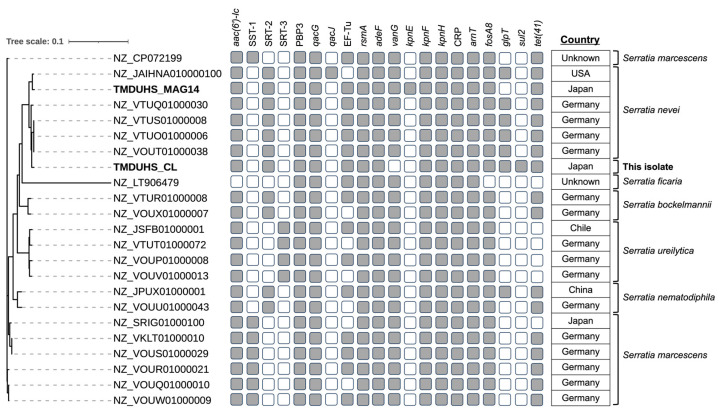
Phylogenetic tree of *Serratia* spp. genomes from the metagenome-assembled genome (MAG) and bacterial isolates. The maximum likelihood tree was constructed with 1000 bootstrap replicates. The presence of antimicrobial-resistant genes in these genomes is indicated.

The *Serratia* sp. strain TMDUHS_CL was isolated from the hospital wastewater sample and identified using 16S rRNA gene sequencing (Figure 6). The phylogenetic analysis revealed a close genetic relationship between TMDUHS_CL and the *S. nevei* cluster, which also included MAG14. The whole-genome sequencing analysis of TMDUHS_CL revealed various ARGs, including *aac(6′)-Ic*, SRT-2, PBP3, *qacG*, EF-Tu, *rsmA*, *adeF*, *kpnF*, *kpnH*, CRP, *armT*, *fosA8*, *glpT*, *sul2*, and *tet(41)*, as identified according to CARD. The antimicrobial susceptibility profiles of the isolates are listed in Table S6. These isolates acquired resistance to ampicillin, cefazolin, and colistin and exhibited intermediate resistance to ampicillin/sulbactam, based on the Clinical and Laboratory Standards Institute guidelines.

## 3. Discussion

The presence and activation of AMR are major public health hazards because of the critical harm AMR causes to human health and the associated difficult-to-treat infections [14,15,16]. In the present study, we investigated the distribution and function of ARGs in MAG- and plasmid-derived sequences from hospital wastewater using metagenomic and metatranscriptomic analyses. Metatranscriptomic data are highly informative and can distinguish highly active bacteria and strongly expressed genes from those that are inactively present in the environment, helping to identify noteworthy ARBs and ARGs [11]. In the present study, we identified active ARGs on a plasmid and successfully isolated an ARB that could be functional in the sample based on the expression profiles of ARGs, highlighting the effectiveness of metatranscriptome analysis and providing insights into AMR dynamics in aquatic environments.

Hospitals are considered to be a major source of numerous pollutants, and hospital wastewater contains a high concentration of residual antibiotics and ARGs [4]. The antibiotic residues in an aquatic environment can select ARB, even at low concentrations, leading to a unique distribution of ARGs in the microbial community [17,18]. In the present study, several antibiotics with different antimicrobial mechanisms were identified in hospital wastewater, of which ampicillin, one of the β-lactams, was predominantly detected. Previous studies have demonstrated stress-regulated expression of specific ARGs, such as those involved in antimicrobial target protection systems within the microbiome [19,20]. It has also been proposed that antibiotic compounds reflect the trends of ARG abundances [21]. We observed high frequencies of acrR and soxS, which control the β-lactam and fluoroquinolone efflux systems, and the presence of folP, which is associated with sulfamethoxazole resistance [22,23]. Antimicrobial compounds in hospital effluents play major roles in the overexpression of ARGs [10]. Our metagenomic and metatranscriptomic data revealed different gene distribution patterns, which may reflect the influence of antimicrobial exposure on microbial communities. Compared with the DNA counts, the number of mRNAs of *omp* encoding OMP (Figure 3D) was markedly increased, which may be due to the high concentration of ampicillin in hospital wastewater. While OMPs exhibit resistance to β-lactam owing to genetic mutations, certain OMPs also reportedly regulate other OMPs by increasing their expression levels, leading to β-lactam resistance [24]. This observation suggests an association between the expression levels of ARGs and antimicrobials. However, a variety of non-antibiotic substances with antimicrobial activity from hospitals affect the proportion and transmission of ARGs in wastewater [25,26], emphasizing the need for a multifaceted understanding of environmental water, functional attributes, and microbial communities.

Although ARGs were both present and actively transcribed in the hospital wastewater, the ratio of mRNA to DNA varied among these genes, with notable differences observed in the normalized counts between sequences derived from MAGs and those from plasmids. In the metagenome binning process used to construct MAGs, plasmid sequences present in the assembled genome were reportedly lost from the genomes of plasmid-containing bacteria [27]. In the present study, tetracycline-resistance genes, several of which have been found in transmissible elements [28], were observed in plasmid-derived sequences. Multidrug resistance genes linked to antimicrobial efflux and reduced permeability to antimicrobials were most frequently detected in sequences originating from both MAGs and plasmids, whereas the mRNA/DNA ratio was higher in plasmid-derived sequences (1.73) than in MAG-derived sequences (0.32). The ARGs of plasmid-derived sequences were actively transcribed in comparison with the MAG-derived sequences, such as *mexS* (5.49 vs. 0.84), *acrR* (3.20 vs. 0.50), and *mexR* (1.55 vs. 0.25), which are involved in multidrug resistance through efflux pump regulation. These findings indicate that genes associated with multidrug resistance are widely distributed in hospital wastewater, particularly those with plasmid-derived sequences that function in the environment. However, these data present certain limitations in bioinformatic analysis.

We used several verification tools to identify plasmid-associated contigs; nonetheless, genes typically found on chromosomes, such as *gyrA* and *parC* [29], were detected, albeit at a low rate, in sequences originating from plasmids. This finding suggests that the accurate detection of plasmid-derived sequences remains challenging. Transformation and transduction have recently been shown to play roles in environmental horizontal gene transfer, whereas plasmid conjugation has the greatest influence on ARG dissemination [30]. Accordingly, analytical approaches are required to accurately assess genetic factors other than plasmids that influence the spread of ARGs. Notably, ARGs identified using CARD included not only ARGs that confer AMR through acquisition but also genes that cause AMR through mutations, regardless of whether the mutation is present or not. For example, a high prevalence of an ARG-encoding EF-Tu, an essential component of bacteria known to be resistant to pulvomycin owing to genetic mutations [31], has been reported. Nevertheless, its effect on phenotypic AMR in aquatic environments remains unclear. Thus, understanding changes in the abundance and expression levels of ARGs requires individual interpretation to identify the features of interest in the environment. Further improvements in bioinformatic tools and the accurate evaluation of ARGs are required to provide reliable metagenomic and metatranscriptomic information.

The MAG approach, based on genome assembly and binning, enables the characterization of numerous microbial genomes related to AMR [8]. In a previous study analyzing hospital effluents using metagenomics, numerous bacterium-derived contigs were found to carry ARGs within microbial communities, suggesting a potential link between ARB and ARGs [8]. Metatranscriptomic research explores the full range of mRNA molecules of an organism to assess the resistome more accurately, unlike metagenomic research, which focuses solely on DNA [32]. We conducted both metagenome and metatranscriptome analyses to comprehensively understand gene expression levels in each MAG. We then focused on MAG14 (*S. nevei*), which exhibited high mRNA levels of ARGs relative to DNA levels. The genus Serratia shows high genetic plasticity, enabling it to thrive in various environments, such as soil, water, and hospitals [33]. Recently, multi-drug-resistant Serratia sp. has emerged as a global pathogen, causing infections and outbreaks in vulnerable individuals [33]. *Serratia nevei* was classified as a species related to *S. marcescens* and has been reported to exhibit high-level resistance to colistin [34]. Using a culture-based method, *Serratia* sp. isolate obtained from hospital wastewater was found to be resistant to several antibiotics, including ampicillin/sulbactam detected by LC–MS/MS and colistin. Phylogenetic analysis of the whole genomes of the isolate and MAG14 confirmed that the two strains were genetically similar, indicating both belong to the same genus, *Serratia*. These strains differed in the possession patterns of certain ARGs, which was attributed to the precision of genome completeness in the metagenomic analysis and extrachromosomal elements [35]. Therefore, the detection of active ARB through metatranscriptomic data analysis can enhance our understanding of remarkable phenotypic AMR. Additionally, it supports the conclusion that the bacteria identified at the genetic level are actively present in the environment, validating the genomic analysis results.

The main limitation of this study is that it was conducted at a single institution based on a single wastewater sample. The types of drug-resistant bacteria that are prevalent and the types of drugs prescribed in hospitals vary from region to region, which may be discharged directly into the wastewater. Although this study measured antibiotic compounds as environmental pressure in hospital wastewater, a variety of non-antibiotic chemicals and agents may cause microbial selection in a complex water matrix [36]. Therefore, data should be collected from a wide range of areas using this integrated metagenomic and metatranscriptomic approach, which was validated by comparison with culture-based methods, in order to confirm its broader applicability.

In conclusion, the combined analysis of metagenomic and metatranscriptomic data offers a comprehensive understanding of environmental AMR. As it is not practical to monitor all ARGs within the environment as part of the “One Health Approach,” establishing a reasonable and appropriate AMR surveillance system is necessary, especially for identifying critical ARGs that require monitoring in aquatic environments where the current status of AMR is unclear [37,38]. The present study indicated that diverse bacteria, including human pathogens, are hosts to ARGs in hospital wastewater, and their ARG profiles differed at the DNA and mRNA levels. Our transcribed gene investigation also revealed core ARB and ARGs that can be used as targets for AMR surveillance, contributing to a reliable assessment of the emergence and spread of AMR in the environment. Further initiatives to evaluate the clinical effect of AMR and its influence on the ecosystem linked to hospital wastewater are required for sustainably balanced public health.

## 4. Materials and Methods

### 4.1. Hospital Wastewater Collection and Sample Preparation

A single 5 L sample of hospital wastewater was taken at the effluent point of all the untreated wastewater from Institute of Science Tokyo Hospital, which is a teaching hospital with over 800 beds, on 12 December 2022, and was processed to extract nucleic acids within 1 h. The sample was concentrated, and an aliquot was used for DNA and RNA extraction. The DNA for 16S rRNA gene metabarcoding and shotgun metagenomic analysis was extracted using a DNeasy PowerSoil Pro Kit (Qiagen, Hilden, Germany), following the manufacturer’s instructions. The RNeasy PowerWater Kit (Qiagen) was used to recover total RNA from the sample according to the supplier’s protocol. For LC–MS/MS analysis, the wastewater sample was stored under dark conditions at 4 °C and processed within 3 days.

### 4.2. LC–MS/MS Analysis

The target components included amoxicillin, ampicillin, ceftriaxone, cefcapene pivoxil, imipenem, meropenem, doripenem, sulbactam, erythromycin, azithromycin, lincomycin, doxycycline, levofloxacin, sulfamethoxazole, and trimethoprim. These were selected either because they are critically important antibiotics for human medicine or because they have been previously detected in wastewater in Japan [39]. The reagent cefcapene pivoxil was purchased from the Tokyo Chemical Industry (Tokyo, Japan); the other reagents were purchased from FUJIFILM Wako Pure Chemical (Osaka, Japan). A solution of imipenem, meropenem, and doripenem was prepared in high-performance LC-grade methanol (Kanto Chemical, Tokyo, Japan) containing 0.1% guaranteed formic acid (FUJIFILM Wako Pure Chemical). The other antibiotics were dissolved in methanol. Carbamazepine-d10 (FUJIFILM Wako Pure Chemical) was used as the internal standard.

The ACQUITY UPLC I-Class and XevoTQ system (Waters, Milford, MA, USA) were used for the LC–MS/MS analysis of imipenem, meropenem, and doripenem, with the analytical conditions detailed in Tables S7 and S8. Five hundred milliliters of wastewater sample were processed using solid-phase extraction with InertSep C18 and InertSep SlimGC cartridges (GL Sciences, Tokyo, Japan), followed by elution solely from InertSep SlimGC with 5 mL of 0.1% formic acid in 50% methanol, and subsequently concentrated to 1 mL under nitrogen flow. The LC–MS/MS analysis of the other antibiotics was conducted using the ACQUITY UPLC H-Class and XevoTQD system (Waters), with the specific analytical parameters listed in Tables S9 and S10. Wastewater (500 mL), adjusted to pH < 3 with formic acid, was prepared for solid-phase extraction using a Sep-Pak Plus PS-2 and Oasis HLB Plus LP Extraction Cartridge (Waters), followed by elution by backflushing with 4 mL of high-performance LC-grade acetonitrile (FUJIFILM Wako Pure Chemical). The volume was further concentrated to 0.1 mL under nitrogen flow and then adjusted to 0.5 mL with ultrapure water.

### 4.3. 16S rRNA Gene Metabarcoding Analysis

The V3–V4 region of the 16S rRNA gene was sequenced using the MiSeq platform with MiSeq Reagent Kit V3 (Illumina, San Diego, CA, USA), as previously described by Caporaso et al. [40]. The first PCR was conducted in a reaction mixture containing KAPA HiFi HotStart (Kapa Biosystems, Wilmington, MA, USA) and primer set (5′-TCGTCGGCAGCGTCAGATGTGTATAAGAGACAGCCTACGGGNGGCWGCAG-3′ and 5′-GTCTCGTGGGCTCGGAGATGTGTATAAGAGACAGGACTACHVGGGTATCTAATCC-3′) under the following amplification conditions: initial denaturation at 95 °C for 3 min, 32 cycles of denaturation at 95 °C for 30 s, annealing at 55 °C for 30 s, and extension at 72 °C for 30 s, with a final extension at 72 °C for 5 min. The 2nd PCR was performed using KAPA HiFi HotStart and primer set (5′- AATGATACGGCGACCACCGAGATCTACAC-(index sequence)-TCGTCGGCAGCGTC-3′ and 5′-CAAGCAGAAGACGGCATACGAGAT-(index sequence)-GTCTCGTGGGCTCGG-3′) under the following amplification conditions: initial denaturation at 95 °C for 3 min, 8 cycles of denaturation at 95 °C for 30 s, annealing at 55 °C for 30 s, and extension at 72 °C for 30 s, with a final extension at 72 °C for 5 min. The DADA2 pipeline [41] was used for noise filtering and clustering of the raw reads. The taxonomic assignment of the amplicon sequence variants was compared using the SILVA reference database [12].

### 4.4. Shotgun Metagenomic Analysis

Shotgun metagenomic data were generated using the DNBSEQ-G400RS platform and MGIEasy FS DNA Library Prep Set (MGI Tech, Shenzhen, China), according to the supplier’s instructions. DNA Nanoballs were prepared after ligation of adapters to fragmented genomic DNA and 8 cycles of PCR amplification. Fastp v0.20.1 [42] was used to remove adaptor contaminants and low-quality reads. The output reads were paired and assembled using Megahit v1.2.9 [43]. To recover individual genomes, the binning modules Metabat1 v0.32.5 [44], Metabat2 v2.12.1 [45], and Maxbin2 v2.2.7 [46] were used to reconstruct MAGs from the assemblies. The results of each method were integrated using DasTool v1.1.1 [47] for the quality assessment of MAGs. The bacterial species in each MAG were identified using GTDB Toolkit Classify v2.1.0 [48]. Prokka v1.14.6 [49] and eggNOG-mapper online version [50] were used for the annotation of each MAG. ARGs were predicted using the CARD [13]. To predict plasmid-derived sequences from assembled contigs using Megahit v1.2.9 [43], we used the plasmidVerify tool [51] to verify the identified putative plasmids and compared the sequences with those in the PLSDB [52]. Plasmid incompatibility groups were identified using PlasmidFinder v2.0 [53].

### 4.5. Shotgun Metatranscriptomic Analysis

Shotgun metatranscriptomic sequencing was conducted using DNBSEQ-T7RS (MGI Tech), the MGIEasy RNA Directional Library Prep Set (MGI Tech), and the NEBNext rRNA Depletion Kit (bacteria) (New England Biolabs, Beverly, MA, USA), according to the supplier’s instructions. DNA Nanoballs were prepared after ligation of adapters to reverse-transcribed RNA and 16 cycles of PCR amplification. Quality filtering and rRNA removal were performed on the output reads using Fastp v0.20.1 [42] and RiboDetector v0.2.4 [54], respectively. The expression level of each MAG and plasmid-derived sequence was analyzed using FeatureCount v2.0.1 [55] and normalized using TPM [56]. The MAGs and plasmid-derived contigs based on the metagenomic data were normalized per unit length using the TPM scale, and their expression levels were compared relative to the amount of DNA present in the sample. Spearman’s rank correlation analysis using GraphPad Prism ver. 8.3.0 (GraphPad Software, San Diego, CA, USA) was applied to determine the relationships between ARG abundance and the expression, as well as MAG- and plasmid-based data.

### 4.6. Isolation and Whole-Genome Sequencing of Serratia sp.

As *Serratia nevei*, found in the metagenome analysis, has been reported to be resistant to colistin [34], the hospital wastewater sample stored at 4 °C was plated on bromothymol blue agar supplemented with 4 μg/mL colistin and incubated under aerobic conditions at 37 °C. Bacterial colonies with distinct morphologies were identified using 16S rRNA gene sequencing [57]. For the strain identified as *Serratia* sp., genomic DNA was extracted using the MagAttract HMW DNA Kit (Qiagen) according to the manufacturer’s instructions. DNA libraries for short reads were prepared using the DNA Prep (M) Tagmentation Kit (Illumina, San Diego, CA, USA), and sequencing was performed on a NovaSeq 6000 platform (Illumina). Raw short reads were quality-filtered using Fastp v0.20.1 [42]. Unicycler v0.4.8 [58] was used to assemble the trimmed reads. The genome sequences of 21 *Serratia* spp., as reported in previous studies [34], were obtained from the GenBank genome database. Antimicrobial-resistant genes were detected using CARD [13]. Genomes annotated using Prokka v1.13 [49] were subjected to pan-genome analysis using Roary v3.13.0 [59]. A maximum likelihood phylogenetic tree was constructed using IQ-TREE v2.2.0.3 [60], and the tree was visualized using iTOL v6 [61].

### 4.7. Antimicrobial Susceptibility Testing

A broth microdilution test was conducted to determine the minimum inhibitory concentrations (MICs) of the antimicrobials on commercially available dry plates (Eiken Chemical, Tokyo, Japan) to clarify the resistance profile of AMR isolates. Twenty antibiotics, ampicillin, piperacillin, ampicillin/sulbactam, piperacillin/tazobactam, cefazolin, ceftriaxone, ceftazidime, cefepime, aztreonam, imipenem, meropenem, doripenem, colistin, gentamicin, amikacin, tobramycin, minocycline, ciprofloxacin, levofloxacin, sulfamethoxazole/trimethoprim, and fosfomycin, commonly used for Enterobacterales infection, were tested and the MIC results were interpreted based on the Clinical and Laboratory Standards Institute document, M100-Ed32 [62].

## Data Availability

Raw 16S rRNA amplicon sequencing, metagenomic, and metatranscriptomic data included in this study have been deposited in the GenBank database under the accession numbers SRR28388879, SRR28388881, and SRR28388880, respectively. The accession numbers of the assembled genomes of MAG14 and the *Serratia* isolate are SAMN40370724 and SAMN40276084, respectively.

## Supplementary Materials

The following supporting information can be downloaded at: https://www.mdpi.com/article/10.3390/antibiotics13121122/s1, Figure S1: The list of metagenome-assembled genomes at the species level in hospital wastewater.; Figure S2: Correlation heatmap illustrating the relationship between the incompatibility group and presence of antimicrobial-resistant genes (ARGs) in plasmids identified using PlasmidFinder.; Table S1: Summary for 16S rRNA gene metabarcoding, shotgun metagenomic, and shotgun metatranscriptomic sequencing.; Table S2. 16S rRNA gene amplicon sequencing result at the genus level.; Table S3. Summary for metagenome-assembled genomes (MAGs).; Table S4. Occurrence and expression levels of antimicrobial resistance genes (ARGs) from metagenome-assembled genomes (MAGs).; Table S5. Occurrence and expression levels of antimicrobial resistance genes (ARGs) from plasmid-derived sequences.; Table S6. Antimicrobial susceptibility profile of Serratia isolate.; Table S7. The analytical conditions of liquid chromatography-tandem mass spectrometry (LC-MS/MS) for imipenem, meropenem, and doripenem.; Table S8. Tandem mass spectrometry (MS/MS) parameters for imipenem, meropenem, and doripenem.; Table S9. The analytical conditions of liquid chromatography-tandem mass spectrometry (LC-MS/MS) for antimicrobials other than imipenem, meropenem, and doripenem.; Table S10. Tandem mass spectrometry (MS/MS) parameters for antimicrobials other than imipenem, meropenem, and doripenem.
