# Supplementary material for: Metatranscriptomic Analysis Reveals Actively Expressed Antimicrobial-Resistant Genes and Their Hosts in Hospital Wastewater
Source: Antibiotics (Basel). 2024 Nov 23;13(12):1122. doi: 10.3390/antibiotics13121122 (PMC11672649; doi:10.3390/antibiotics13121122)
Supplement: Supplementary file 1 [file antibiotics-13-01122-s001.zip › Supplementary/Supplementary Figure.pdf]

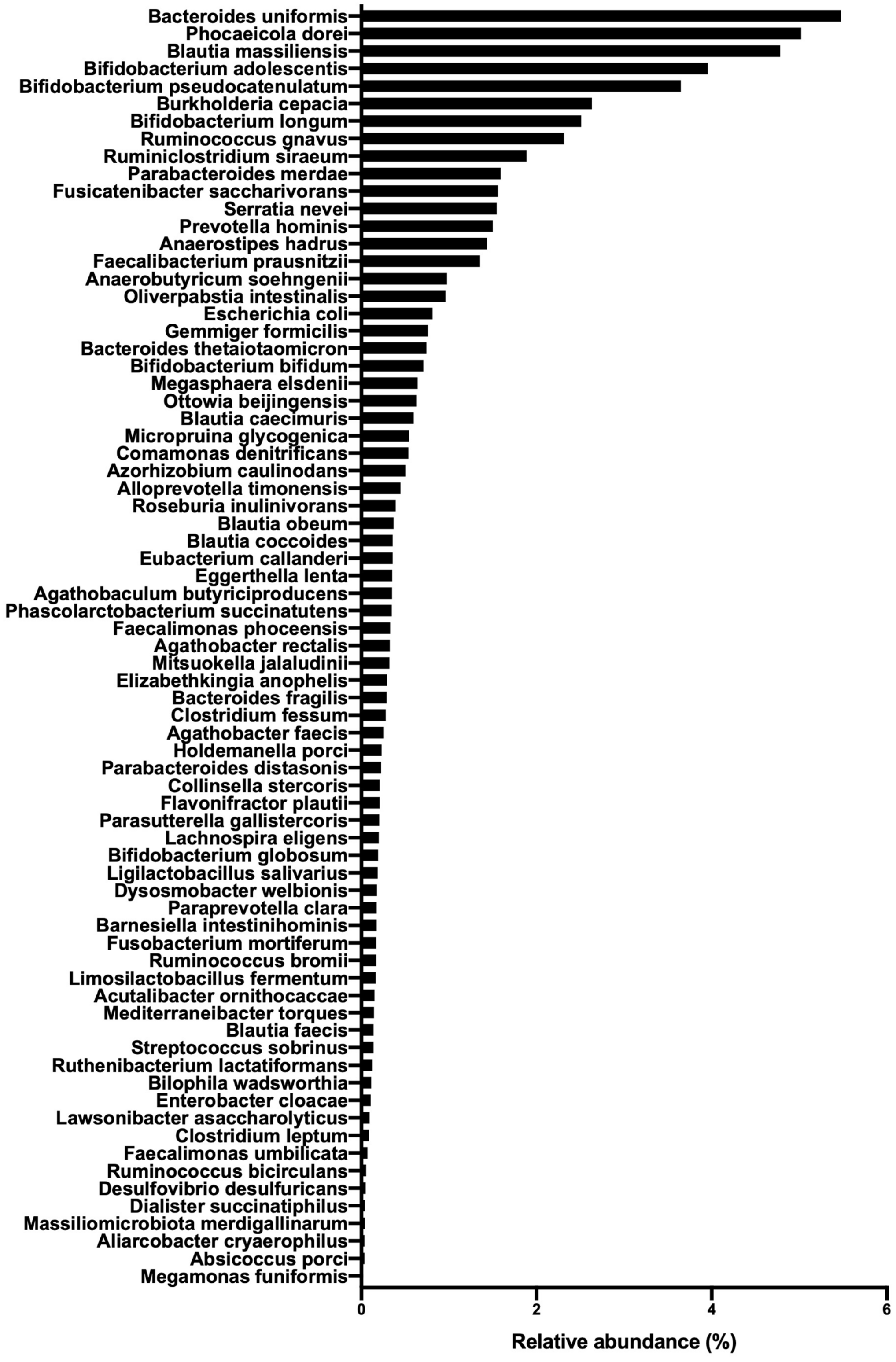

Figure S1. The list of metagenome-assembled genomes at the species level in hospital wastewater.

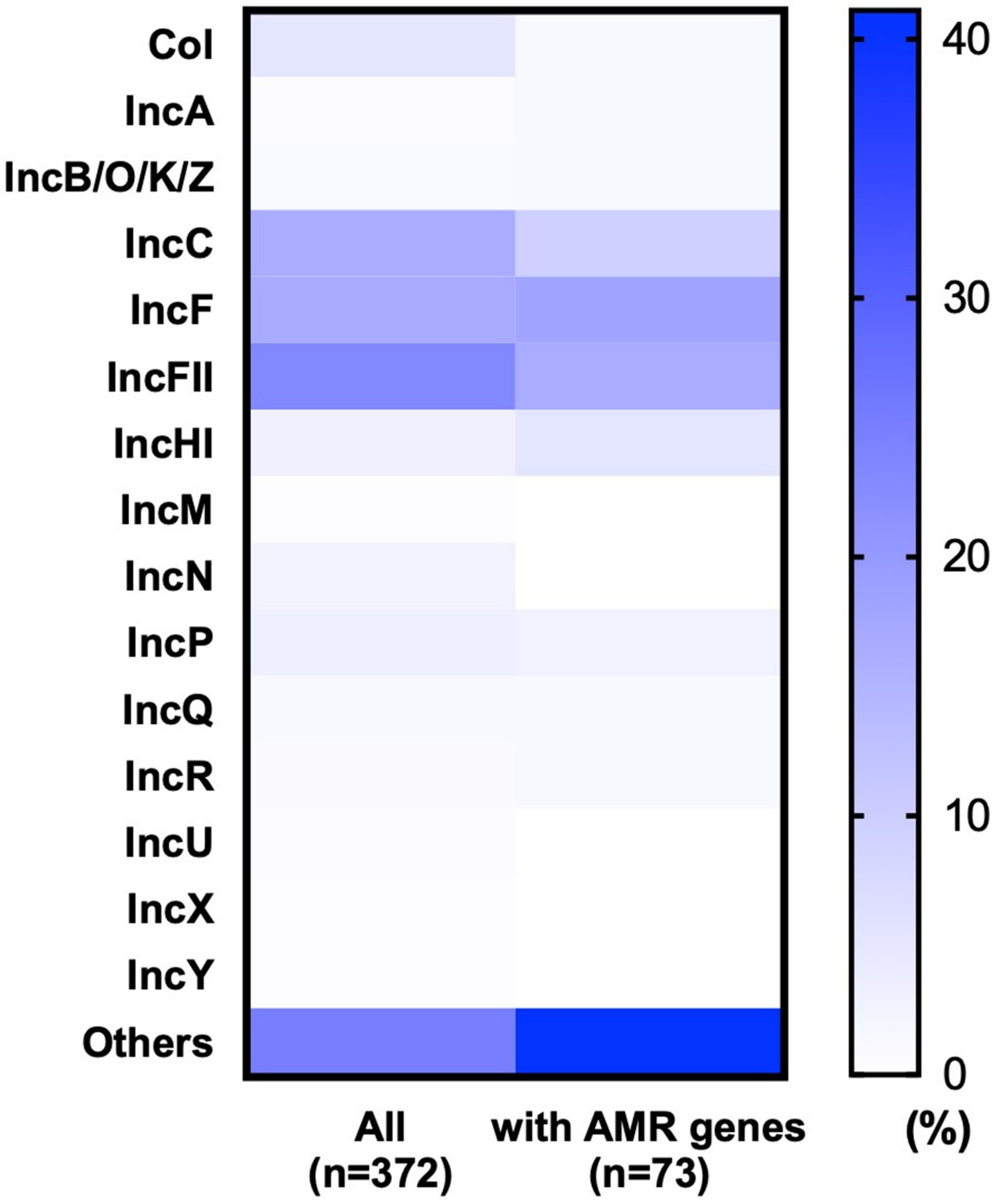

Figure S2. Correlation heatmap illustrating the relationship between the incompatibility group and presence of antimicrobial-resistant genes (ARGs) in plasmids identified using PlasmidFinder.
